# A New Nanomaterial Based on Extracellular Vesicles Containing Chrysin-Induced Cell Apoptosis Through Let-7a in Tongue Squamous Cell Carcinoma

**DOI:** 10.3389/fbioe.2021.766380

**Published:** 2021-11-26

**Authors:** Zhijing Yang, Da Liu, Hengzong Zhou, Boqiang Tao, Lu Chang, Huimin Liu, Haoming Luo, Dongxu Wang, Weiwei Liu

**Affiliations:** ^1^ Department of Oral and Maxillofacial Surgery, Hospital of Stomatology, Jilin University, Changchun, China; ^2^ Laboratory Animal Center, College of Animal Science, Jilin University, Changchun, China; ^3^ Department of Pharmacy, Changchun University of Chinese Medicine, Changchun, China

**Keywords:** extracellular vesicles, gold nanoparticles, let-7a, cell apoptosis, tongue squamous cell carcinoma

## Abstract

Although the therapeutic strategy showed significant improvement, the therapeutic effect was poor on metastases in tongue squamous cell carcinoma (TSCC) which is the most malignant tumor found in the head and neck. Chrysin, similar to the flavonoids, plays an antitumor role by regulating the expression of ncRNAs in many kinds of cancers. Compared to flavonoids, gold nanoparticles (AuNPs) provide a novel insight into inhibiting cancer cell growth via photothermal therapy (PPT) which is irradiated by near-infrared radiation (NIR). However, most flavonoids and AuNPs lack specificity of tumor *in vivo*. The extracellular vesicles (EVs) which were abundant with ncRNAs are isolated from the cellular supernatant fluid and have the ability to carry drugs or nanoparticles to improve specificity. In the present study, we aimed to synthesize a new nanomaterial based on EVs containing chrysin and analyzed cell apoptosis in TSCC cells. Our results demonstrated that EVs-chrysin were isolated from SCC9 cells that were treated with chrysin. To improve the therapeutic effect, AuNPs were carried by EVs-chrysin (Au-EVs). Compared to BGC823 and HCC-LM3 cells, the uptake of Au-EVs was specific in SCC9 cells. Moreover, Au-EVs combined with NIR enhanced cell apoptosis in TSCC cells. To confirm the role of miRNAs in cell apoptosis, the differentially expressed miRNAs between EVs-Con and EVs-chrysin were screened by RNA-seq. The results revealed that the let-7a-3p family, which acts as the tumor suppressor, was upregulated in EVs-chrysin compared to EVs-Con. Thus, let-7a-3p was screened in the apoptosis pathway that was associated with the p53 protein. Furthermore, compared to the Con group, Au-EVs combined with the NIR group effectively inhibited tumor growth *in vivo* via increasing the expression of let-7a-3p. Together, as a new nanomaterial, Au-EVs induced cell apoptosis and inhibited tumor growth by regulating let-7a-3p expression in TSCC.

## Introduction

Although there have been advances in the therapy and diagnosis of tongue squamous cell carcinoma (TSCC), a type of oral cancer, the 5-year overall survival rate remains low. Therapeutic strategies for TSCC include surgery, radiotherapy, and chemotherapy. In the past decades, traditional Chinese medicine has also been applied in cancer research. Chrysin is present in honey and exhibits anticancer function. Previous reports indicated that chrysin can induce cell apoptosis and inhibit proliferation via ncRNAs in many cancer cells (Zhong et al., 2020; Yufei et al., 2020). Compared to traditional therapeutic strategies, gold nanostructures, such as nanostars (Gao et al., 2015), popcorns (Bhana et al., 2015), and nanoclusters (Yang et al., 2018a), have been widely studied for drug delivery and diagnostics in cancer treatment. Gold nanoparticles (AuNPs), combined with NIR, have also been applied in PPT (Liu et al., 2020; Hirsch et al., 2003). However, AuNPs and chrysin exhibit a lack of specificity with regard to cancers. Our strategy is to use AuNPs with EVs that could specifically accumulate in tongue and squamous cell carcinoma (Li et al., 2018).

Previous data demonstrated that the uptake of EVs is specific to cell type (Lara et al., 2020).Thus, nanomaterials combined with EVs can improve the efficiency in the therapeutics of cancer. There is evidence that nanoparticles combined with EVs function as drug carriers targeted to cancer cells. (Yong et al., 2019).Moreover, EVs are endogenous and can carry non-coding RNAs (ncRNAs), mRNA, drugs, or peptides to have anticancer effects (Valadi et al., 2007; Kim et al., 2016). NcRNAs, such as miRNAs, have proven to have a role in cancer development (Di Leva et al., 2014; Huang et al., 2021). MiRNAs have 20–22 nucleotides and are abundant in EVs (Zhang et al., 2015). As tumor suppressors, the let-7a family shows less expression in various cancers, such as breast cancer (Wu et al., 2015). Increasing the expression of let-7a inhibited invasion and migration via the MAPK pathway in prostate cancer cells (Tang et al., 2018). Previous studies suggested that let-7a expression was related to the long non-coding RNA (lncRNA) *H19,* which was associated with cell apoptosis (Kallen et al., 2013; Ghafouri-Fard et al., 2020). However, there is little evidence showing the let-7a expression pattern in Au-EVs and in TSCC cells.

In the present study, chrysin was used to treat TSCC cells and isolated EVs-chrysin. HAuCl_4_ was incubated with EVs-chrysin to form Au-EVs to treat SCC9 cells or tumors with NIR. In addition, the let-7a family was analyzed after the RNA-seq screened between EVs-Con and EVs-chrysin. Our findings indicated that Au-EVs induced apoptosis through let-7a in TSCC.

## Materials and Methods

### Cell Culture and Chrysin Treatment

Human TSCC cell lines SCC9 and CAL27, human gastric cancer cell line BGC823, and human hepatocellular carcinoma cell line HCC-LM3 were cultured in Dulbecco’s modified Eagle medium (DMEM; Gibco) or DMEM/F12, to which was added 10% fetal bovine serum (Gibco). SCC9 and CAL27 cells were treated with 40 μM of chrysin (Yuanye Bio-Technology, Shanghai) for 48 h.

### Preparation of EVs-Chrysin and Au-EVs

The EVs were extracted from the culture medium of SCC9 cells that were treated with chrysin (EVs-chrysin) or PBS (EVs-Con). The culture medium of SCC9 cells with 70% confluency was immediately collected. Then, the cell culture was centrifuged at 300×g for 30 min, at 2000×g for 30 min, and at 12, 000×g for 45 min, and then the supernatant fluid was filtered. Next, the filter liquor was ultracentrifuged (Thermo Scientific) at 110, 000×g for 70 min. The EVs were collected and washed with PBS. The EVs were ultracentrifuged at 110, 000×g for 70 min again. For Au-EVs, the collected EVs that contained chrysin (EVs-chrysin) were incubated with HAuCl_4_ (50 mM, Sigma) at 37°C overnight. The particle size and concentration of EVs-Con and EVs-chrysin were detected using dynamic light scattering (DLS) by nano-flow cytometry (NanoFCM, China). The shape of the EVs-Con, EVs-chrysin, and Au-EVs was determined by transmission electron microscopy (TEM, HITACHI, HT-7800).

### The Detection of EV Proteins

The EVs (40 μl) were diluted and fluorescent-labeled antibodies were added (20 μl, CD9, CD63, CD81, and BGI) at 37°C for 30 min. Then, 1 ml of PBS was added and ultracentrifugated at 4°C, at 110,000×g for 70 min. IgG, CD9, CD63, and CD81 of EVs were detected by NanoFCM (BGI, China).

### Preparation of PKH26-Labeled EVs-Chrysin and Au-EVs

The EVs-Con, EVs-chrysin, and Au-EVs were labeled with PKH26. First, 1 μl PKH26 linker and 9 μl diluent C were premixed. Then, the EVs-Con, EVs-chrysin, and Au-EVs (10 and 30 μg) were added to the mixed solution and incubated for 10 min. Next, the EVs-Con, EVs-chrysin, and Au-EVs that were labeled with PKH26 were ultracentrifuged at 100, 000×g for 70 min. Finally, 200 μl PBS was added to resuspend the labeled EVs-Con, EVs-chrysin, and Au-EVs. A laser confocal microscope was used to capture the image.

### Analysis of Uptake of Au-EVs

5×10^4^ of SCC9, BGC823, and HCC-LM3 cells were cultured and incubated with 200 μl–labeled Au-EVs for 15 min or 24 h. The harvested cells were washed with PBS to remove non-incorporated AuEVs. Hoechst was used to label the nuclei. The image was observed using a laser confocal microscope.All experiments were performed in triplicate.

### RNA-Seq Analysis of EVs-Chrysin

The total RNA from EVs-Con and EVs-chrysin was extracted. The EVs were treated with PBS as a con group (EVs-Con). Using DNBSEQ (Beijing Genomics Institute, BGI, China), 264 small RNAs were identified. Quality control was performed on the raw reads to get clean reads. Then, the clean reads (total clean reads, 32M) were aligned to the reference gene sequence. Small RNAs were counted and classified. The differential miRNA expression was determined (Fold Change >0.5, FDR <0.001). Using Dr.Tom software (BGI, China), the Kyoto Encyclopedia of Genes and Genomes (KEGG) pathway and the GO enrichment analysis of differentially expressed miRNAs were determined. The data were submitted to the Gene Expression Omnibus (GEO) database (GSE185562).

### Collection of TSCC Samples

Three patients underwent clinical surgery, and their samples were collected. The TSCC samples were immediately stored in liquid nitrogen for RNA isolation.

### Knockdown and Overexpression of Let-7a

The let-7a-3p sequence is listed in Supplementary Table S1. The let-7a-3p mimics and inhibitor were purchased from GenePharma (Shanghai, China). The SCC9 cells were transfected with the mimic and inhibitor of let-7a-3p for 48 h. Nonspecific siRNA (Nc) was used as the control.

### Analysis of miRNAs Expression

RNAs of TSCC cells and tumors were extracted and cDNAs (cDNA First-Strand Synthesis Kit, TIANGEN, China) were generated. QPCR (quantitative real-time PCR) was performed to analyze the miRNA expression pattern. The primers of miRNAs and apoptosis genes are listed in Supplementary Table S2. The qPCR conditions were as follows: 94°C for 3 min and then 94°C for 10 s after 35 cycles. The annealing was carried out at 59°C for 15 s. The products were extended at 72°C for 30 s. U6 or GAPDH was used as the control. All experiments were performed in triplicate.

### Cell Migration and Invasion Analysis

Cell migration was measured by applying a wound healing assay in the SCC9 cells. The SCC9 cells (5 × 10^5^) were treated with let-7a-3p mimics or inhibitors for 48 h. Then, the cells were scratched and cultured with FBS medium. The scratched area was analyzed at intervals of 12, 24, and 48 h.

The SCC9 cells (3 × 10^4^) were used for cell invasion assays. The cells were transfected with the let-7a-3p mimics or inhibitor. Matrigel matrix (20 μl, BD Biosciences, United States) was added and incubated along with the medium overnight. Crystal violet dye (0.2%, Solarbio, China) was used to stain the SCC9 cells. The stained cells were analyzed using ImageJ software. Each group of experiments was performed in triplicate.

### The Colony Formation Assay

The SCC9 cells (1 × 10^3^) were used for the colony formation assay. The cell colonies were fixed with paraformaldehyde and stained with crystal violet after 8 days. The number of cells were counted using ImageJ. The experiments of the colony formation assay were performed in triplicate.

### Cell Apoptosis Analysis

The let-7a-3p mimics or inhibitor were transfected in SCC9 cells. An annexin V-FITC/PI reagent was used for cell apoptosis analysis. Flow cytometry (BD Biosciences, Franklin Lakes, NJ, United States) was performed to detect apoptosis cells. All the apoptosis experiments were performed in triplicate.

### TUNEL Assay

The SCC9 cells were treated with Au-EVs and Au-EVs with NIR. The Con group was treated with PBS. Then, the cells were fixed with 4% paraformaldehyde. These cells were blocked with PBS containing 1% BSA in the dark for 1 h with TdT and fluorescein-conjugated dUTPs (*In Situ* Cell Death Detection kit; Roche, Germany). DAPI was used to stain the nuclei. A fluorescence microscope was used to capture the images. The TUNEL assay was performed in triplicate.

### Western Blot

The protein was collected using a protein extraction buffer (Beyotime, China). The BCA assay was used to quantify the protein (Tiangen, Beijing, China). The protein separation process used sodium dodecyl sulfate-polyacrylamide gel electrophoresis. The polyvinylidene difluoride (PVDF) membrane was incubated with a p53 antibody (Abcam, ab131442, United States), BAX (Cell Signaling Technology, D2E11, United States), BCL-2 (Cell Signaling Technology, D55G8, United States), caspase-3 (Wanleibio, WL02117, China), and GAPDH (Bioworld, AP0066, United States). HRP-conjugated AffiniPure goat antibodies IgG (Boster, China) were used as a secondary antibody. The ECL Super Signal kit (Pierce, United States) was used to analyze the bands.

### Hematoxylin and Eosin (HE) Staining

The lungs, liver, spleen, kidneys, and heart tissues from the Con, Au-EVs, and Au-EVs + NIR groups were fixed in 4% paraformaldehyde for 48 h. Then, the samples were embedded in paraffin wax and cut into 5-μm sections. The slides were stained with H and E and observed under a light microscope.

### Animals

The female nude mice (N = 20, 6–8 weeks old) were utilized to determine tumor growth *in vivo*. The nude mice were injected with SCC9 cells (8×10^6^) into the left flank and then divided into four groups: the Con, chrysin, Au-EVs, and Au-EVs + NIR groups. The tumors were observed after 7 days. The Con group was treated with saline water. The chrysin group was treated with chrysin (20 mg/kg) each day by intragastric administration. The PKH26-labeled Au-EVs were subcutaneously injected below the tumor on day 8 and day 15. The Au-EVs + NIR group was exposed to NIR (808 nm) after injection (twice, day 8 and day 15), and the tumor growth was observed *in vivo* with a fluorescence imaging system (λex = 530 nm, λem = 600 nm, AniView600, Guangzhou Biolight Biotechnology, China).

### Statistical Analysis

An unpaired Student’s *t*-test was utilized in the present study. Statistical analysis was conducted using GraphPad Prism 5.0 (GraphPad Software, Inc,.). All data were expressed as mean ± SD. A *p-*value <0.05 was considered statistically significant.

## Results

### Synthesis and Characterization of Au-EVs

**FIGURE 1:**
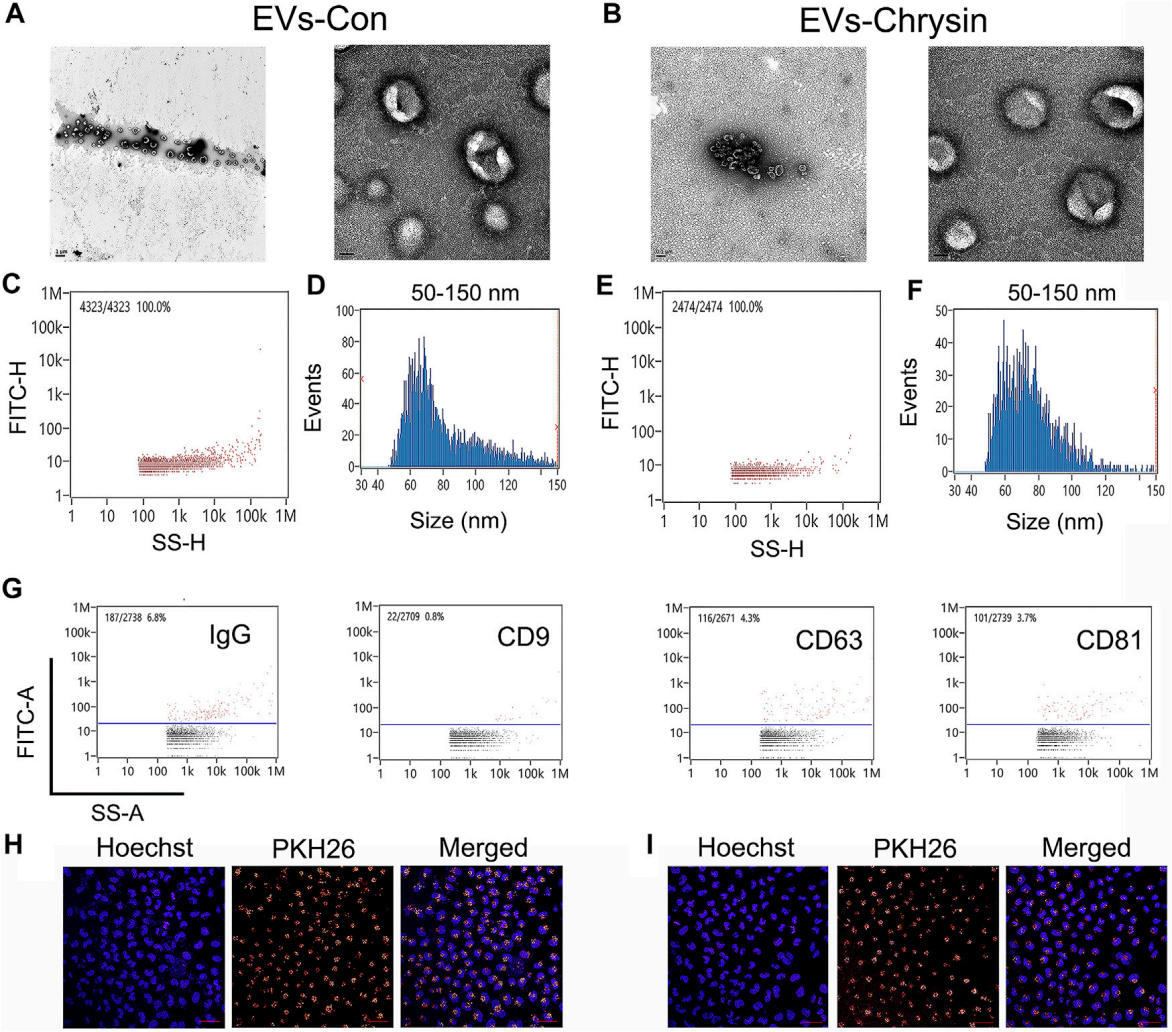
Characterization of cell-derived EVs. Shape of EVs-Con and EVs-chrysin using TEM **(A, B)**. Concentration and size of EVs-Con **(C, D)** and EVs-chrysin **(E, F)** using DLS analysis. Biomarker of EVs-Con and EVs-chrysin was detected using NanoFCM **(G)**. EVs-Con **(H)** and EVs-chrysin **(I)** incubated with PKH26-labeled SCC9 cells. The scale bar was 50 μm. Blue indicated Hoechst, and red indicated PKH26-labeled EVs.

**FIGURE 2:**
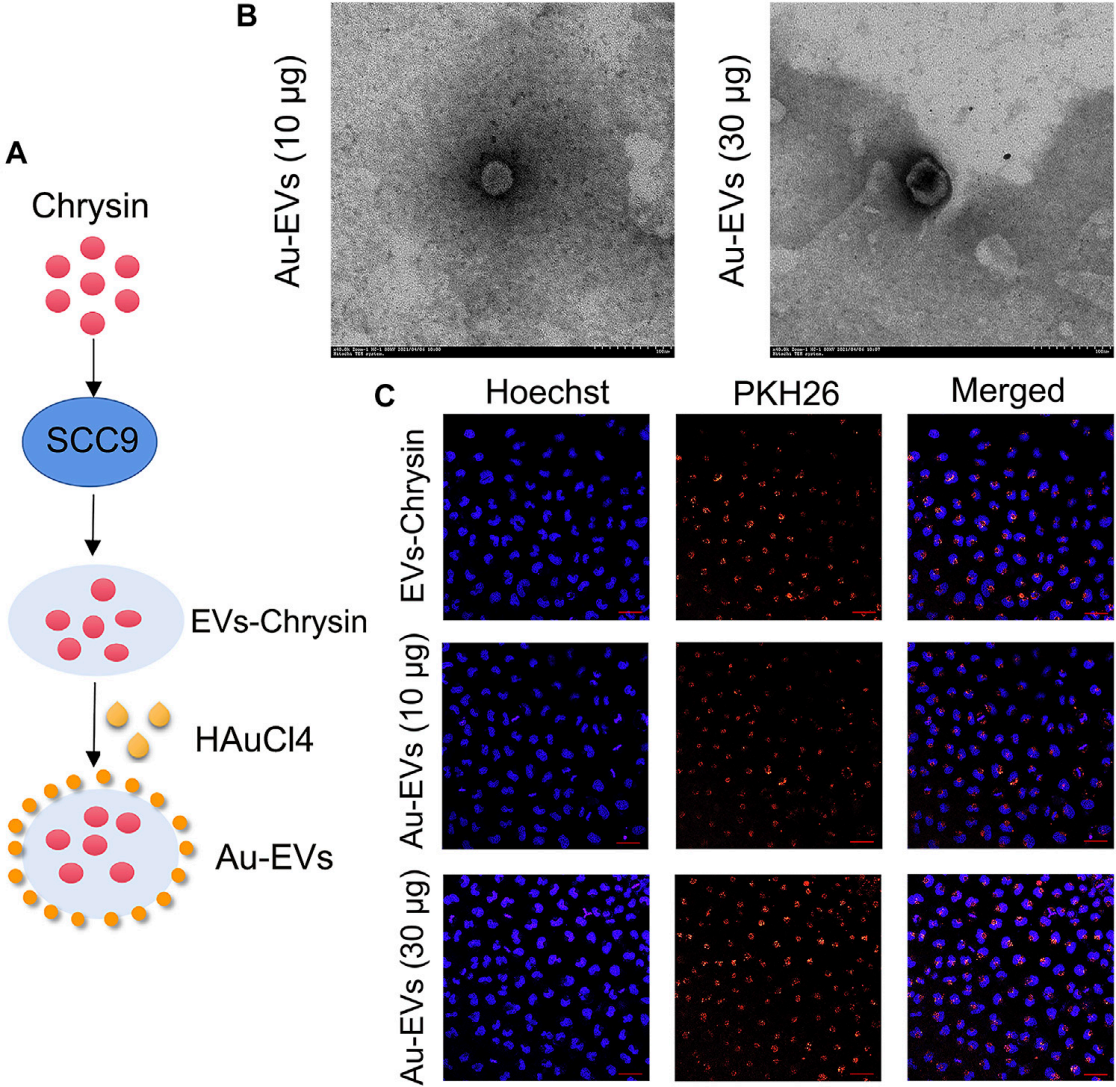
Preparation and synthesis of Au-EVs. Scheme of preparation of Au-EVs which contains chrysin **(A)**. Shape of Au-EVs using TEM **(B)**. EVs-chrysin, Au-EVs (10 μg), and Au-EVs (30 μg) incubated with PKH26-labeled SCC9 cells **(C)**. Scale bar was 50 μm. Blue indicated Hoechst, and red indicated PKH26-labeled EVs.

To obtain EVs, chrysin was treated in SCC9 cells. Our results showed that the shape of the EVs-Con and EVs-chrysin was round (Figures 1A,B). The DLS analysis determined that the size of the EVs-Con and EVs-chrysin was 50–150 nm (Figures 1C–F). The NanoFCM results indicated that CD9, CD63, and CD81 appeared in the EVs-chrysin (Figure 1G). Furthermore, the SCC9 cells were treated with the EVs-Con and the EVs-chrysin. The results showed that the EVs-Con and EVs-chrysin were successfully absorbed by SCC9 cells (Figures 1H,I). These results indicated that the EVs were isolated from SCC9 cells that contained chrysin and are absorbed by SCC9 cells. To improve the antitumor effect of EVs-chrysin, HAuCl_4_ was used to incubate EVs-chrysin and synthesize Au-EVs (Figure 2A). To find the optimal protocol for EVs-chrysin to synthesize Au-EVs, 10 and 30 μg of EVs-chrysin were analyzed. The results showed that AuNPs were self-grown on the surface of EVs-chrysin (both 10 and 30 μg) and formed a new nanomaterial, which was Au-EV (Figure 2B). Similar to EVs-chrysin, Au-EVs were also effectively absorbed by SCC9 cells (Figure 2C). These results indicated that Au-EVs were stably absorbed in SCC9 cells.

### Specific Uptake of Au-EVs and Induced Apoptosis in SCC9 Cells

**FIGURE 3:**
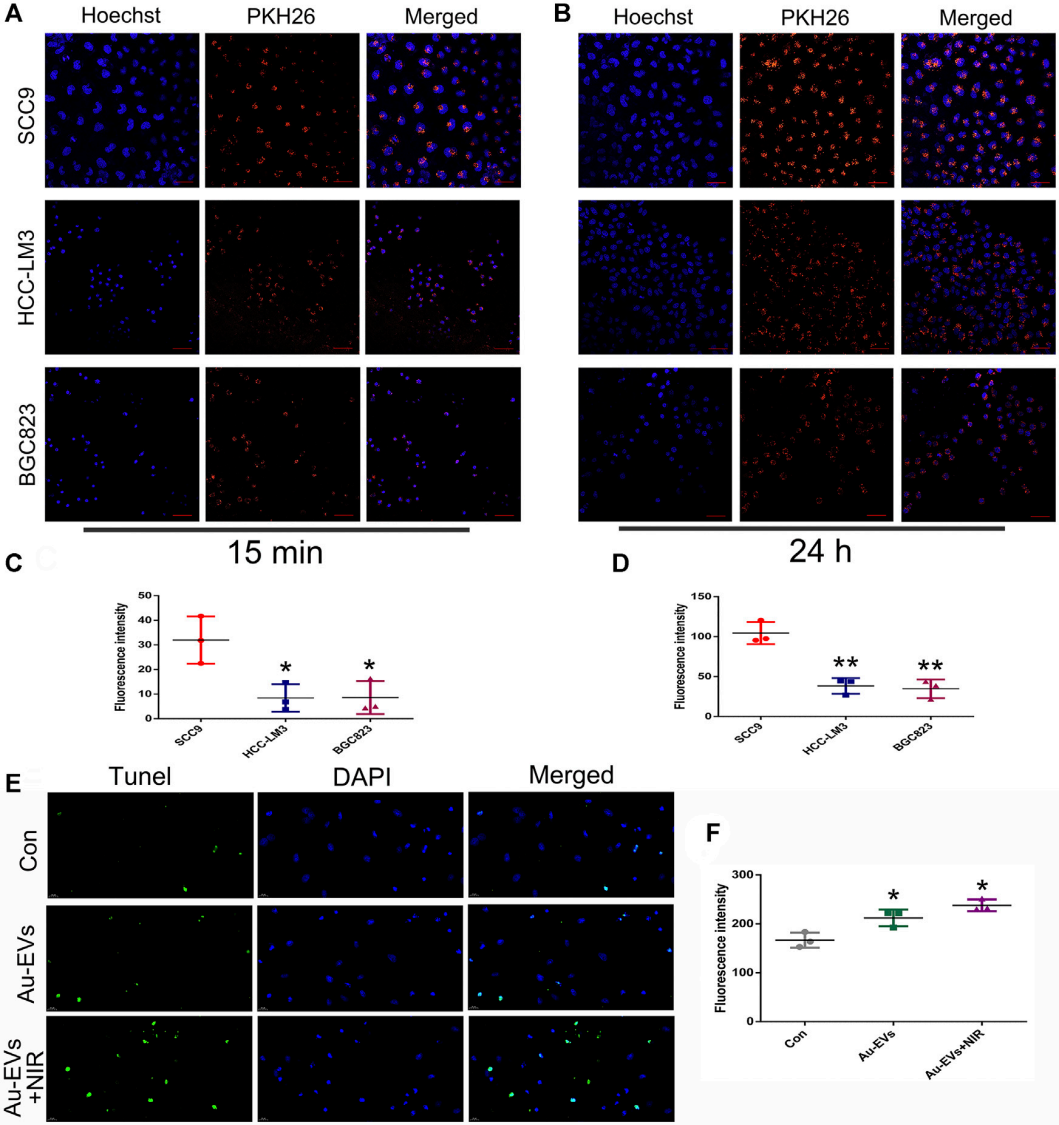
Au-EVs were specifically absorbed and induced cell apoptosis in SCC9 cells. Uptake of Au-EVs by SCC9, HCC-LM3, and BGC823 cells at 15 min **(A)** and 24 h **(B)**. Scale bar was 50 μm. Blue indicated Hoechst, and red indicated PKH26-labeled EVs. Statistical analysis of the fluorescence intensity **(C, D)**. SCC9 cells were treated with Au-EVs and Au-EVs combined with NIR using TUNEL analysis for cell apoptosis **(E)**.Scale bar was 20 μm. Blue indicates DAPI and green indicates TUNEL. * (*p* < 0.05) and ** (*p* < 0.01) indicate statistically significant differences.

To determine whether Au-EVs were specific to the cell type, SCC9, BGC823, and LM3 cells were used. The results showed that, compared to BGC823 and LM3 cells, the uptake of Au-EVs was specific in SCC9 cells (Figures 3A–D). Considering that Au-EVs contain chrysin, the TUNEL assay was used to analyze the cell apoptosis. The results suggested that Au-EVs and Au-EVs combined with NIR induced apoptosis in SCC9 cells (Figures 3E,F). Moreover, irradiation by NIR enhanced apoptosis in SCC9 cells (Figures 3E,F). These results indicated that Au-EVs combined with NIR promote significant apoptosis compared with that of Au-EVs.

### Screening of Differentially Expressed miRNAs in EVs-Chrysin

**FIGURE 4:**
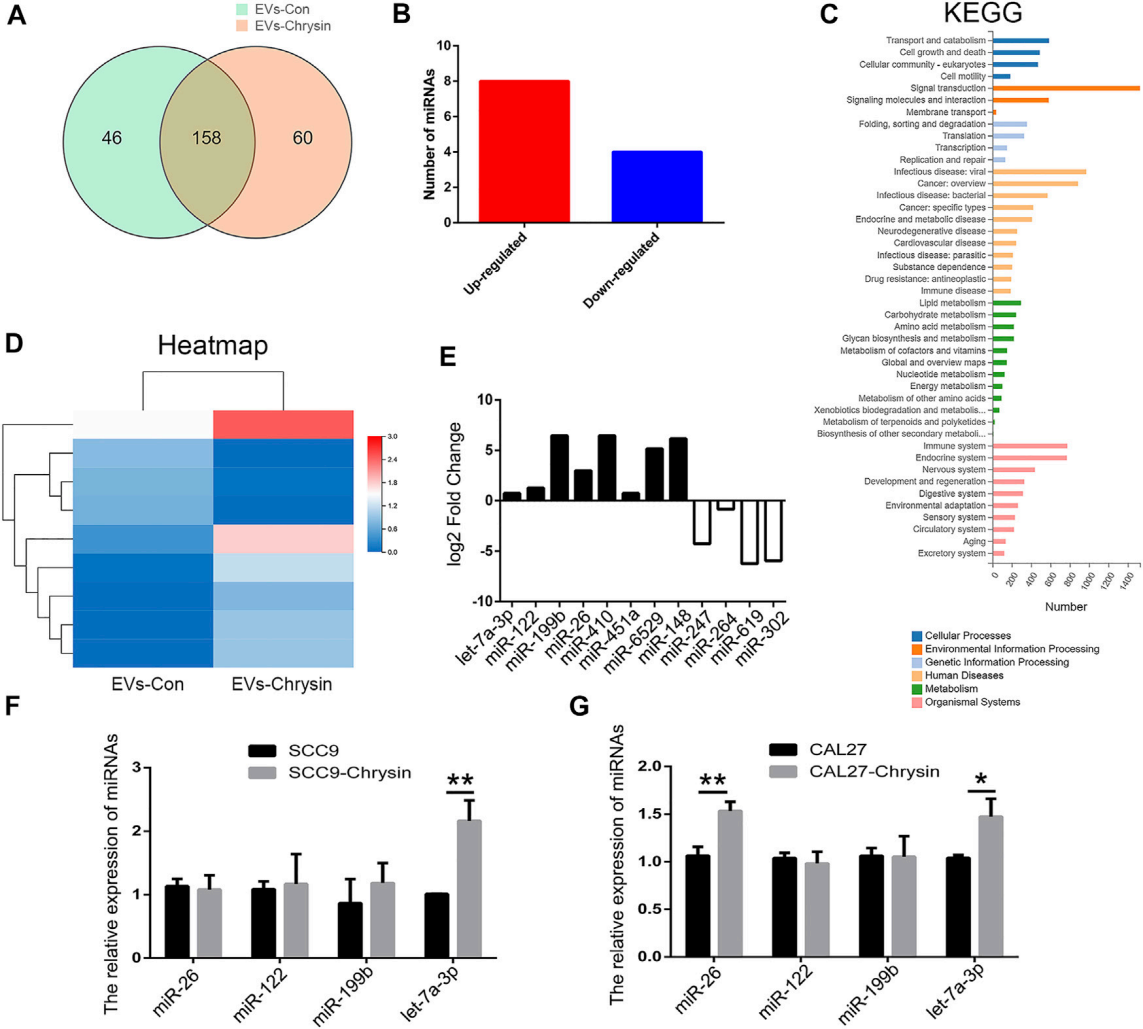
Screening of differentially expressed miRNAs by RNA-seq in EVs. Analysis of differentially expressed genes after chrysin treatment in EVs **(A)**. Identification of differentially expressed miRNAs **(B)**. Heatmap was drawn to show the differentially expressed miRNAs **(C)**. KEGG pathway of the differentially expressed miRNAs **(D)**. Expression of log2 fold change in 12 miRNAs (E). Relative expressions of miR-26, miR-122, miR-199b, and let-7a-3p were analyzed by qPCR after chrysin treatment in SCC9 (F) and CAL27 cells **(G)**. Data are represented as the mean ± SD (*n* = 3). * (*p* < 0.05) and ** (*p* < 0.01) indicate statistically significant differences.

Considering that EVs contain lots of miRNAs that are involved in cell apoptosis, RNA-seq was used to screen differentially expressed miRNAs between EVs-Con and EVs-chrysin. Overall, 158 genes were identified in EVs (Figure 4A). A total of 12 miRNAs were differentially expressed between EVs-Con and EVs-chrysin. Compared to the EVs-Con, 8 miRNAs were significantly upregulated, while 4 were significantly downregulated in EVs-chrysin (Figure 4B). The KEGG pathway and heatmap data indicated that the differentially expressed miRNAs have a role in cell growth and death (Figures 4C,D). Further, we analyzed the log2 fold change in the 8 upregulated miRNAs (let-7a-3p, miR-122, miR-199b, miR-26, miR-410, miR-451a, miR-6529, and miR-148) and the 4 downregulated miRNAs (miR-247, miR-264, miR-619, and miR302) (Figure 4E). To confirm these data, 4 miRNAs (miR-26, miR-122, miR-199b, and let-7a-3p) that were associated with cell growth and death were investigated. The qPCR results indicated that only let-7a-3p was upregulated after chrysin treatment in SSC9 and CAL27 cells (Figures 4F,G). These results suggested that the expression of let-7a-3p that might be involved in cell growth and death is regulated by chrysin.

### Increased Expression of Let-7a Induced Cell Apoptosis

**FIGURE 5:**
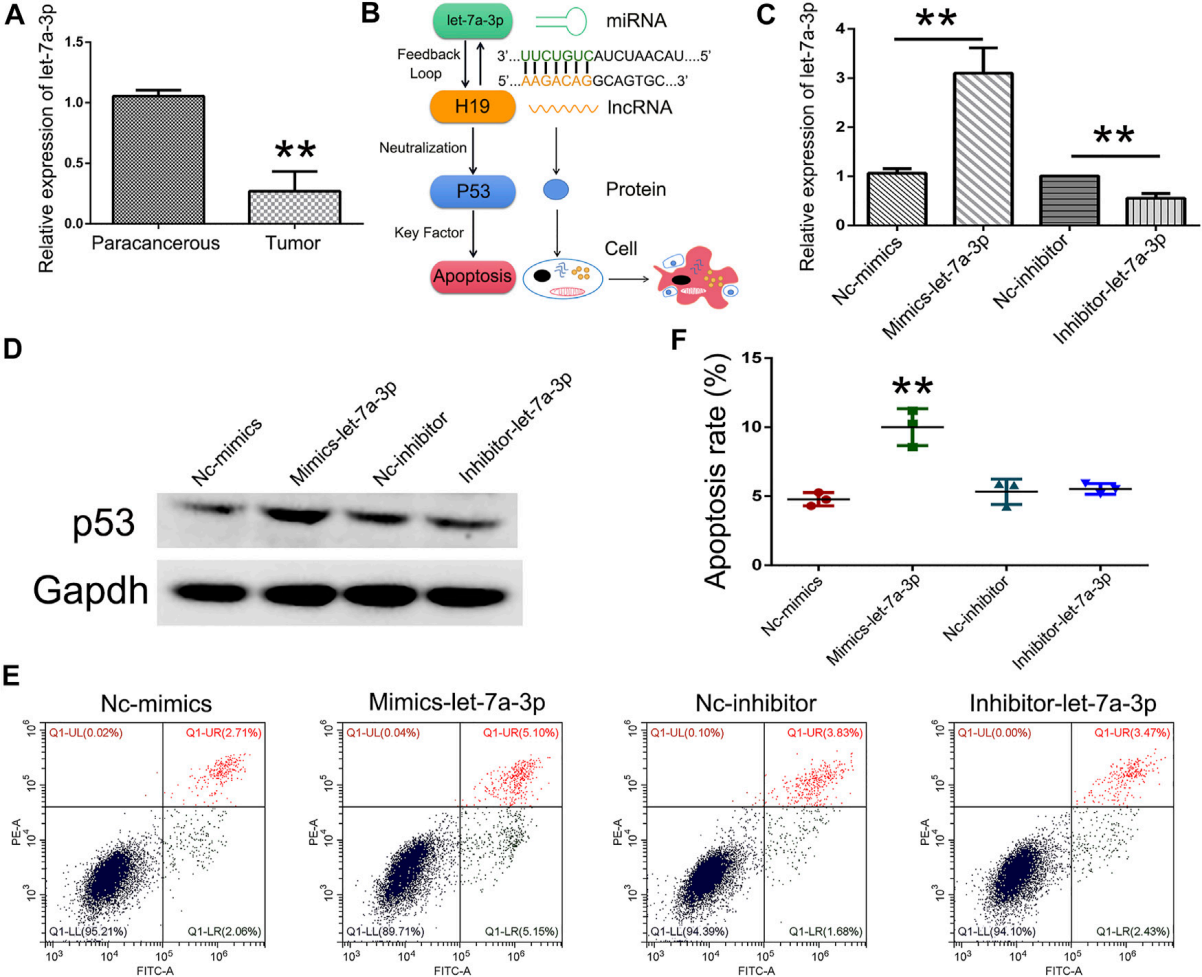
Overexpression of let-7a-3p induced cell apoptosis. Relative expression of let-7a-3p in TSCC patients (*N* = 3) using qPCR **(A)**. Schematic representations of let-7a-3p induced cell apoptosis **(B)**. Relative expression of let-7a-3p in Nc-mimics, mimics-let-7a-3p, Nc-inhibitor, and inhibitor-let-7a-3p groups using qPCR **(C)**. Expression of p53 protein in Nc-mimics, mimics-let-7a-3p, Nc-inhibitor, and inhibitor-let-7a-3p groups using Western blot **(D)**. Cell apoptosis was analyzed after transfection with mimics-let-7a-3p and inhibitor-let-7a-3p in SCC9 cells **(E)**. Statistical analysis of the percentage of cell apoptosis **(F)**. ** (*p* < 0.01) indicate statistically significant differences.

**FIGURE 6:**
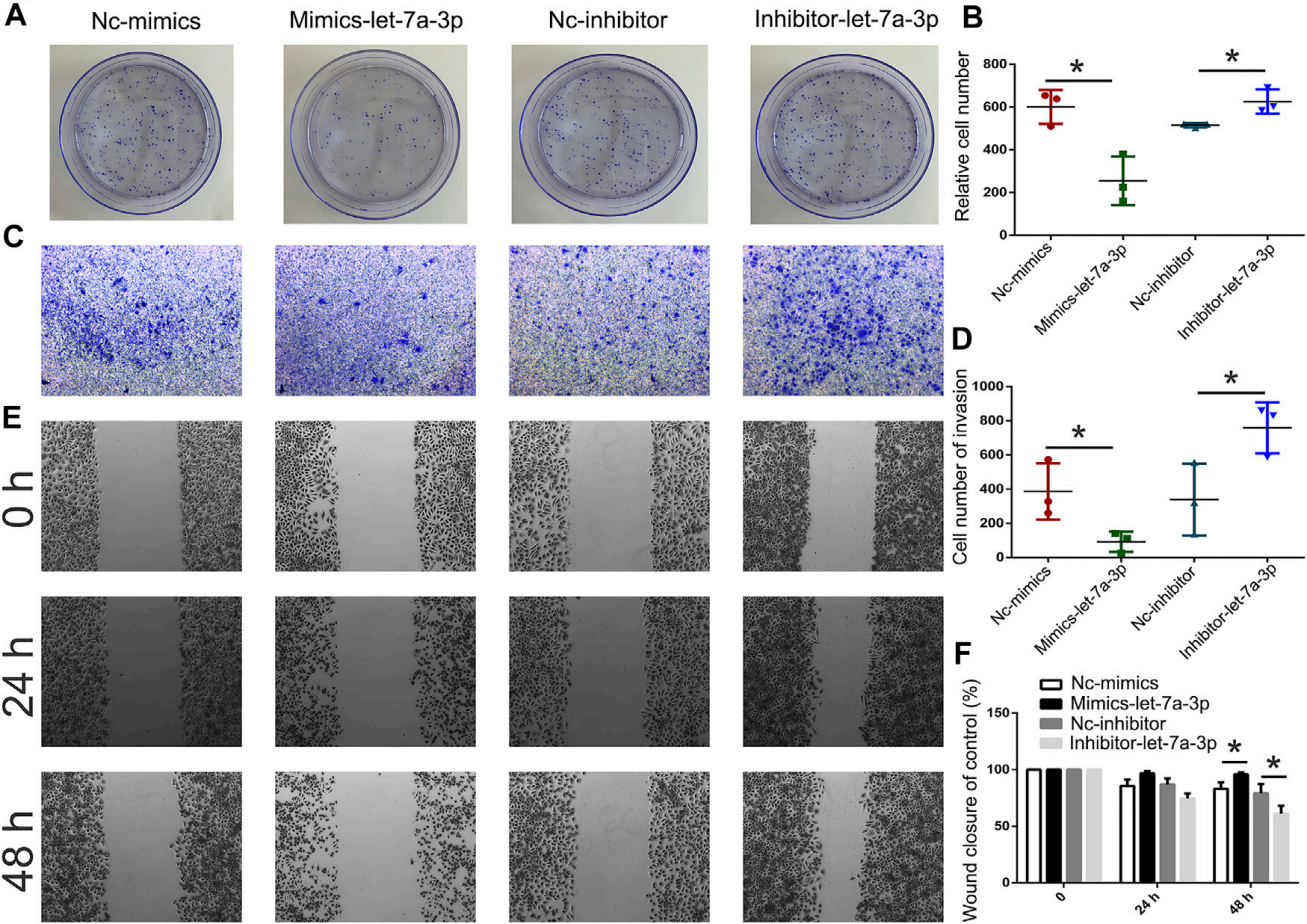
Analysis of cell invasion and migration after transfection with mimics-let-7a and inhibitor-let-7a-3p in SCC9 cells. Cell invasion was analyzed using the colony formation assay after transfection with mimics-let-7a-3p and inhibitor-let-7a-3p in SCC9 cells **(A, B)**. Cell invasion was analyzed using Transwell assay **(C, D)**. Cell migration was analyzed using wound healing assay **(E, F)**. * (*p* < 0.05) indicate statistically significant differences.

**FIGURE 7:**
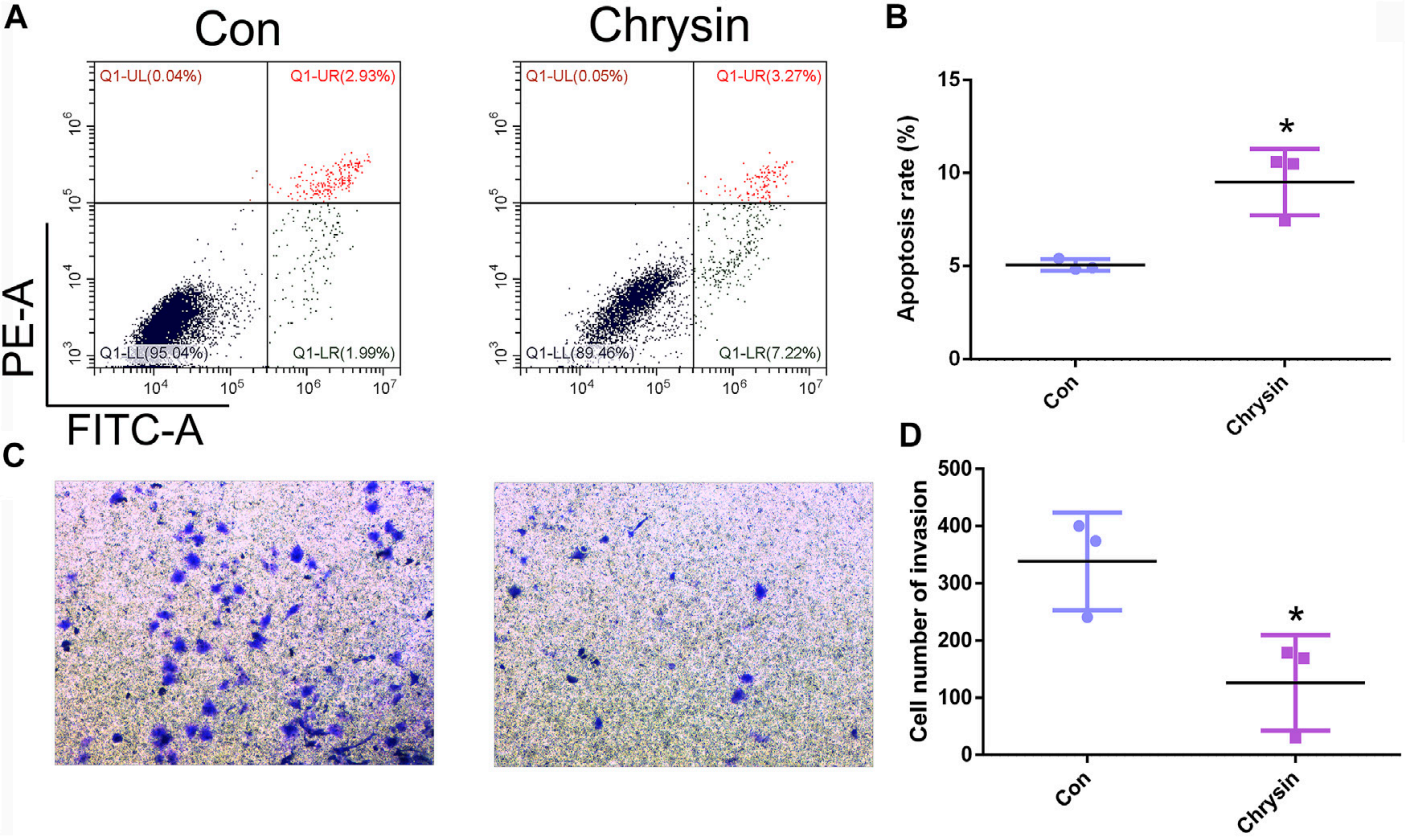
Chrysin induced apoptosis and inhibited invasion in SCC9 cells. Cell apoptosis was analyzed after chrysin treatment in SCC9 cells **(A, B)**. Analysis of cell invasion after chrysin treatment **(C, D)**. * (*p* < 0.05) indicate statistically significant differences.

To analyze the expression pattern of let-7a in TSCC patients, qPCR was performed. The results revealed that let-7a-3p showed less expression in the tumor than the paracancerous tissue (Figure 5A). To determine whether let-7a has a role in cell apoptosis (Figure 5B), overexpression and knockdown expression of let-7a-3p were used. The qPCR and Western blot results indicated that mimics of let-7a-3p increased the expression of the p53 protein, which was a key factor in the cell apoptosis pathway (Figures 5C,D; Supplementary Figure S1). Moreover, increased expression of let-7a-3p induced apoptosis in SCC9 cells (Figures 5E,F). In addition, the overexpression of let-7a-3p inhibited cell invasion (Figures 6A–D). Reduced expression of let-7a-3p promotes migration in SCC9 cells (Figures 6E,F). To confirm the let-7a-3p expression pattern, the SCC9 cells were treated with chrysin. The results showed that chrysin induced cell apoptosis and inhibited invasion (Figure 7). These results indicated that chrysin induced apoptosis and suppressed invasion via let-7a-3p in SCC9 cells.

### Au-EVs Inhibited Tumor Growth *In Vivo*

**FIGURE 8:**
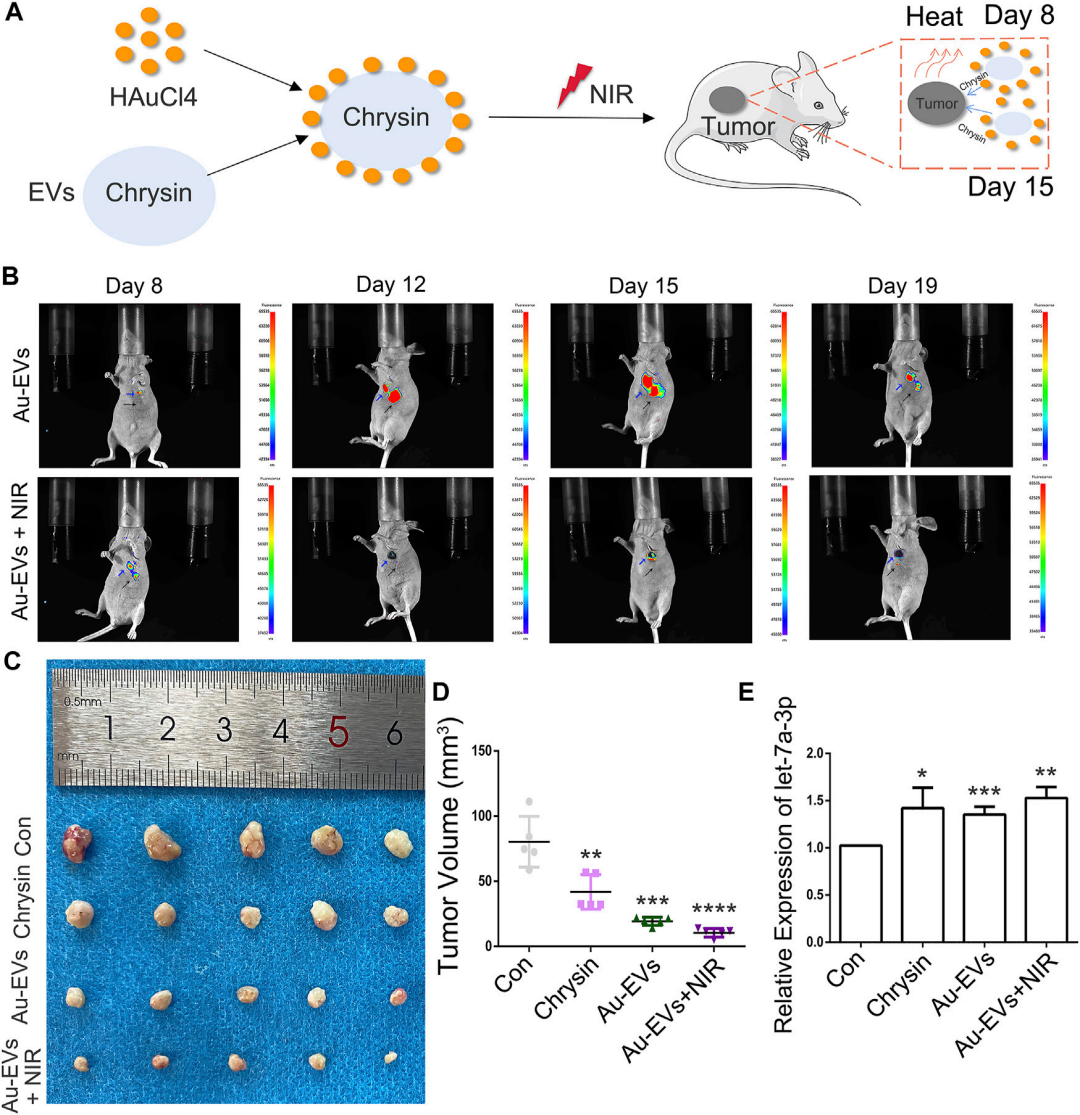
Au-EVs combined with NIR inhibited tumor growth *in vivo.* Schematic representations of Au-EVs injected into nude mice **(A)**. Fluorescence-intense analysis after injection of Au-EVs using *in vivo* imaging **(B)**. Au-EVs were labeled with PKH26. Blue arrow indicated tumor tissues. Black arrow indicated injected sites. Tumor morphology **(C)** and volume **(D)**. Expression pattern of let-7a-3p in the tumors of mice after injection of Au-EVs **(E)**. * (*p* < 0.05), ** (*p* < 0.01), *** (*p* < 0.001), and **** (*p* < 0.0001) indicate statistically significant differences.

To investigate the antitumor effect of Au-EVs *in vivo,* SCC9 cells were injected into nude mice. After 7 days, Au-EVs were injected below the tumor and irradiated with NIR in the nude mice for tumor growth analysis at day 8 and day 15 (Figure 8A). The results suggest that the Au-EVs could move toward the tumor. Moreover, the fluorescence intensity of the Au-EVs increased in a time-dependent manner. In addition, NIR irradiation could quench the fluorescence of the Au-EVs (Figure 8B). At day 21, the tumors were collected. As expected, Au-EVs combined with NIR significantly inhibited tumor growth and did not alter others organs *in vivo* (Figures 8C,D; Supplementary Figure S2). Moreover, the qPCR results indicated that, compared to the Con group, the expression of let-7a-3p was increased in the chrysin and Au-EV groups (Figure 8E). These results demonstrated that Au-EVs mediated PPT effectively and inhibited tumor growth *in vivo.*

## Discussion

In this study, EVs were isolated from SCC9 cells, and our data showed that the EVs exhibited the same size and shape in accordance with B16 EVs and neural stem cell EVs (Lara et al., 2020; Rong et al., 2019). Considering that AuNPs and chrysin have a lack of specificity, EVs were used to contain chrysin and carry AuNPs. EVs-chrysin were incubated with HAuCl_4_ to form Au-EVs which improved the antitumor effect via PPT (Supplementary Figure S3). A previous report indicated that EVs combined with AuNPs are effective against tumors with PPT (Zhang et al., 2019a). In addition, EVs have a higher stability, biocompatibility and biodegradability, lower toxicity, and immunogenicity than other synthesized nanoparticles (Su et al., 2021). Moreover, EVs derived from bone marrow mesenchymal stem cells combined with AuNPs can cross the blood–brain barrier and target neuronal cells (Perets et al., 2019). Evidence suggested that tumor cells selectively absorbed their own EVs, which confirmed our data in SCC9, BGC823, and LM3 cells (Kim et al., 2017). There is evidence that, when combined with NIR, this can accelerate the release of antitumor drugs from EVs and promote cell apoptosis (Zhang et al., 2019a). Our results suggested that the uptake of Au-EVs was specific in SCC9 cells and that Au-EVs combined with NIR enhanced cell apoptosis. EVs were abundant in miRNAs, which have the ability to affect cell growth, invasion, migration, and apoptosis in cancer development (Jurj et al., 2020). An increasing number of miRNAs that are related to cancer development have been studied in EVs (Liu et al., 2019). In the present study, 12 differentially expressed miRNAs were screened by RNA-seq between EVs-Con and EVs-chrysin. An upregulated expression of let-7a-3p was observed in EVs-chrysin. In a previous result, let-7a-3p could induce cell apoptosis through the competitively regulated lncRNA *H19* (Yang et al., 2018b). Moreover, reduced expression of *H19* could increase p53 protein expression in lung cancer cells (Hao et al., 2017). Indeed, p53 protein was a key factor in the cell apoptosis pathway (Paek et al., 2016). In addition, reduced expression of let-7a stimulates cell invasion, migration, and proliferation by targeting MDM4 (Zhang et al., 2019b). Our results demonstrated that the overexpression of let-7a-3p induced apoptosis and inhibited invasion in SCC9 cells. These results indicated that let-7a-3p in EVs-chrysin was involved with cell apoptosis, which was associated with the p53 protein.

To analyze the effect of Au-EVs *in vivo*, PKH26-labeled Au-EVs were used in nude mice. Our data showed that Au-EVs preferentially accumulated in tumor tissues. Moreover, PKH26-labeled Au-EVs have stably existed *in vivo*. Interestingly, combined with NIR, the PKH26-labeled Au-EVs were quenched, which might accelerate chrysin release. A previous report indicated that chrysin inhibited tumor growth *in vivo.* Our results demonstrated that when combined with NIR, Au-EVs effectively inhibited tumor growth *in vivo*. In addition, the let-7a-3p expression of the tumor increased after Au-EVs were injected *in vivo*, which was in accordance with a previous report (Balzeau et al., 2017). Our results suggested that Au-EVs have a high efficiency and can be accurately targeted to inhibit tumor growth *in vivo*.

In summary, as a new nanomaterial, the uptake of Au-EVs was specific in SCC9 cells. Combined with NIR, Au-EVs have effectively enhanced cell apoptosis. Let-7a-3p was screened by RNA-seq in EVs-chrysin and the overexpression of let-7a-3p induced cell apoptosis. Moreover, Au-EVs with NIR significantly inhibited tumor growth *in vivo.* Our results provided a valuable nanomaterial to improve the targeting of AuNPs and are potentially the optimal therapy against TSCC.

## Data Availability

The datasets presented in this study can be found in online repositories. The names of the repository/repositories and accession number(s) can be found below:https://www.ncbi.nlm.nih.gov/geo/query/acc.cgi?acc=GSE185562.
