# Supplementary material for: A New Nanomaterial Based on Extracellular Vesicles Containing Chrysin-Induced Cell Apoptosis Through Let-7a in Tongue Squamous Cell Carcinoma
Source: Front Bioeng Biotechnol. 2021 Nov 26;9:766380. doi: 10.3389/fbioe.2021.766380 (PMC8661124; doi:10.3389/fbioe.2021.766380)
Supplement: Supplementary file 1 [file Table1.docx]

**Table**

**Table S1 S****equence of miR-let7a-3p and *H19***

| Gene | Sequence |
| --- | --- |
| miR-let7a-3p | CUAUACAAUCUACUGUCUUUC |
| H19 | AGTTAGAAAAAGCCCGGGCTAGGACCGAGGAGCAGGGTGAGGGAGGGGGTGGGATGGGTGGGGGGTAACGGGGGAAACTGGGGAAGTGGGGAACCGAGGGGCAACCAGGGGAAGATGGGGTGCTGGAGGAGAGCTTGTGGGAGCCAAGGAGCACCTTGGACATCTGGAGTCTGGCAGGAGTGATGACGGGTGGAGGGGCTAGCTCGAGGCAGGGCTGGTGGGGCCTGAGGCCAGTGAGGAGTGTGGAGTAGGCGCCCAGGCATCGTGCAGACAGGGCGACATCAGCTGGGGACGATGGGCCTGAGCTAGGGCTGGAAAGAAGGGGGAGCCAGGCATTCATCCCGGTCACTTTTGGTTACAGGACGTGGCAGCTGGTTGGACGAGGGGAGCTGGTGGGCAGGGTTTGATCCCAGGGCCTGGGCAACGGAGGTGTAGCTGGCAGCAGCGGGCAGGTGAGGACCCCATCTGCCGGGCAGGTGAGTCCCTTCCCTCCCCAGGCCTCGCTTCCCCAGCCTTCTGAAAGAAGGAGGTTTAGGGGATCGAGGGCTGGCGGGGAGAAGCAGACACCCTCCCAGCAGAGGGGCAGGATGGGGGCAGGAGAGTTAGCAAAGGTGACATCTTCTCGGGGGGAGCCGAGACTGCGCAAGGCTGGGGGGTTATGGGCCCGTTCCAGGCAGAAAGAGCAAGAGGGCAGGGAGGGAGCACAGGGGTGGCCAGCGTAGGGTCCAGCACGTGGGGTGGTACCCCAGGCCTGGGTCAGACAGGGACATGGCAGGGGACACAGGACAGAGGGGTCCCCAGCTGCCACCTCACCCACCGCAATTCATTTAGTAGCAGGCACAGGGGCAGCTCCGGCACGGCTTTCTCAGGCCTATGCCGGAGCCTCGAGGGCTGGAGAGCGGGAAGACAGGCAGTGCTCGGGGAGTTGCAGCAGGACGTCACCAGGAGGGCGAAGCGGCCACGGGAGGGGGGCCCCGGGACATTGCGCAGCAAGGAGGCTGCAGGGGCTCGGCCTGCGGGCGCCGGTCCCACGAGGCACTGCGGCCCAGGGTCTGGTGCGGAGAGGGCCCACAGTGGACTTGGTGACGCTGTATGCCCTCACCGCTCAGCCCCTGGGGCTGGCTTGGCAGACAGTACAGCATCCAGGGGAGTCAAGGGCATGGGGCGAGACCAGACTAGGCGAGGCGGGCGGGGCGGAGTGAATGAGCTCTCAGGAGGGAGGATGGTGCAGGCAGGGGTGAGGAGCGCAGCGGGCGGCGAGCGGGAGGCACTGGCCTCCAGAGCCCGTGGCCAAGGCGGGCCTCGCGGGCGGCGACGGAGCCGGGATCGGTGCCTCAGCGTTCGGGCTGGAGACGAGGCCAGGTCTCCAGCTGGGGTGGACGTGCCCACCAGCTGCCGAAGGCCAAGACGCCAGGTCCGGTGGACGTGACAAGCAGGACATGACATGGTCCGGTGTGACGGCGAGGACAGAGGAGGCGCGTCCGGCCTTCCTGAACACCTTAGGCTGGTGGGGCTGCGGCAAGAAGCGGGTCTGTTTCTTTACTTCCTCCACGGAGTCGGCACACTATGGCTGCCCTCTGGGCTCCCAGAACCCACAACATGAAAGAAATGGTGCTACCCAGCTCAAGCCTGGGCCTTTGAATCCGGACACAAAACCCTCTAGCTTGGAAATGAATATGCTGCACTTTACAACCACTGCACTACCTGACTCAGGAATCGGCTCTGGAAGCTAGAGGAACCAGACCTCATCAGCCCAACATCAAAGACACCATCGGAACAGCAGCGCCCGCAGCACCCACCCCGCACCGGCGACTCCATCTTCATGGCCACCCCCTGCGGCGGACGGTTGACCACCAGCCACCACATCATCCCAGAGCTGAGCTCCTCCAGCGGGATGACGCCGTCCCCACCACCTCCCTCTTCTTCTTTTTCATCCTTCTGTCTCTTTGTTTCTGAGCTTTCCTGTCTTTCCTTTTTTCTGAGAGATTCAAAGCCTCCACGACTCTGTTTCCCCCGTCCCTTCTGAATTTAATTTGCACTAAGTCATTTGCACTGGTTGGAGTTGTGGAGACGGCCTTGAGTCTCAGTACGAGTGTGCGTGAGTGTGAGCCACCTTGGCAAGTGCCTGTGCAGGGCCCGGCCGCCCTCCATCTGGGCCGGGTGACTGGGCGCCGGCTGTGTGCCCGAGGCCTCACCCTGCCCTCGCCTAGTCTGGAAGCTCCGACCGACATCACGGAGCAGCCTTCAAGCATTCCATTACGCCCCATCTCGCTCTGTGCCCCTCCCCACCAGGGCTTCAGCAGGAGCCCTGGACTCATCATCAATAAACACTGTTACAGCAAAAAAAAAAAAAAAA |

**Table S2 Primers for qPCR analysis**

| Genes | Annealing (°C) | Primer sequences (5’→3’) |
| --- | --- | --- |
| *miR-Let-7a-3p* | 60 | F:ACACTCCAGCTGGGCTATACAATCTACTG  R: TGGTGTCGTGGAGTCG  RT:CTCAACTGGTGTCGTGGAGTCGGCAATTCAGTTGAGGAAAGACA |
| *mir-26b-5p* | 60 | F:ACACTCCAGCTGGGTTCAAGTAATTCAGG  R:TGGTGTCGTGGAGTCG  RT:CTCAACTGGTGTCGTGGAGTCGGCAATTCAGTTGAGACCTATCC |
| *mir-122-3p* | 60 | F:ACACTCCAGCTGGGAACGCCATTATCACAC  R:TGGTGTCGTGGAGTCG  RT:CTCAACTGGTGTCGTGGAGTCGGCAATTCAGTTGAGTATTTAGT |
| *mir-199b-5p* | 60 | F:ACACTCCAGCTGGGCCCAGTGTTTAGACTAT  R:TGGTGTCGTGGAGTCG  RT:CTCAACTGGTGTCGTGGAGTCGGCAATTCAGTTGAGGAACAGAT |
| *U6* | 60 | F:GCTTCGGCAGCACATATACTAAAAT  R:CGCTTCACGAATTTGCGTGTCAT  RT:CGCTTCACGAATTTGCGTGTCAT |
| *GAPDH* | 60 | F:TGGTATCGTGGAAGGACTCA  R:GGGCCATCGACAGTCTTC |
| *TP53* | 60 | F:GGCTCTGACTGTACCACCATCCA  R:GGCACAAACACGCACCTCAAAG |
| *BAX* | 60 | F:CCTTTTCTACTTTGCCAGCAAAC  R:GAGGCCGTCCCAACCAC |
| *BCL2* | 60 | F:ATGTGTGTGGAGAGCGTCAACC  R:TGAGCAGAGTCTTCAGAGACAGCC |
| *CASPASE3* | 60 | F:CTCCACAGCACCTGGTTATT  R:AAATTCAAGCTTGTCGGCATAC |
