# Supplementary figures and images for: A New Nanomaterial Based on Extracellular Vesicles Containing Chrysin-Induced Cell Apoptosis Through Let-7a in Tongue Squamous Cell Carcinoma
Source: Front Bioeng Biotechnol. 2021 Nov 26;9:766380. doi: 10.3389/fbioe.2021.766380 (PMC8661124; doi:10.3389/fbioe.2021.766380)

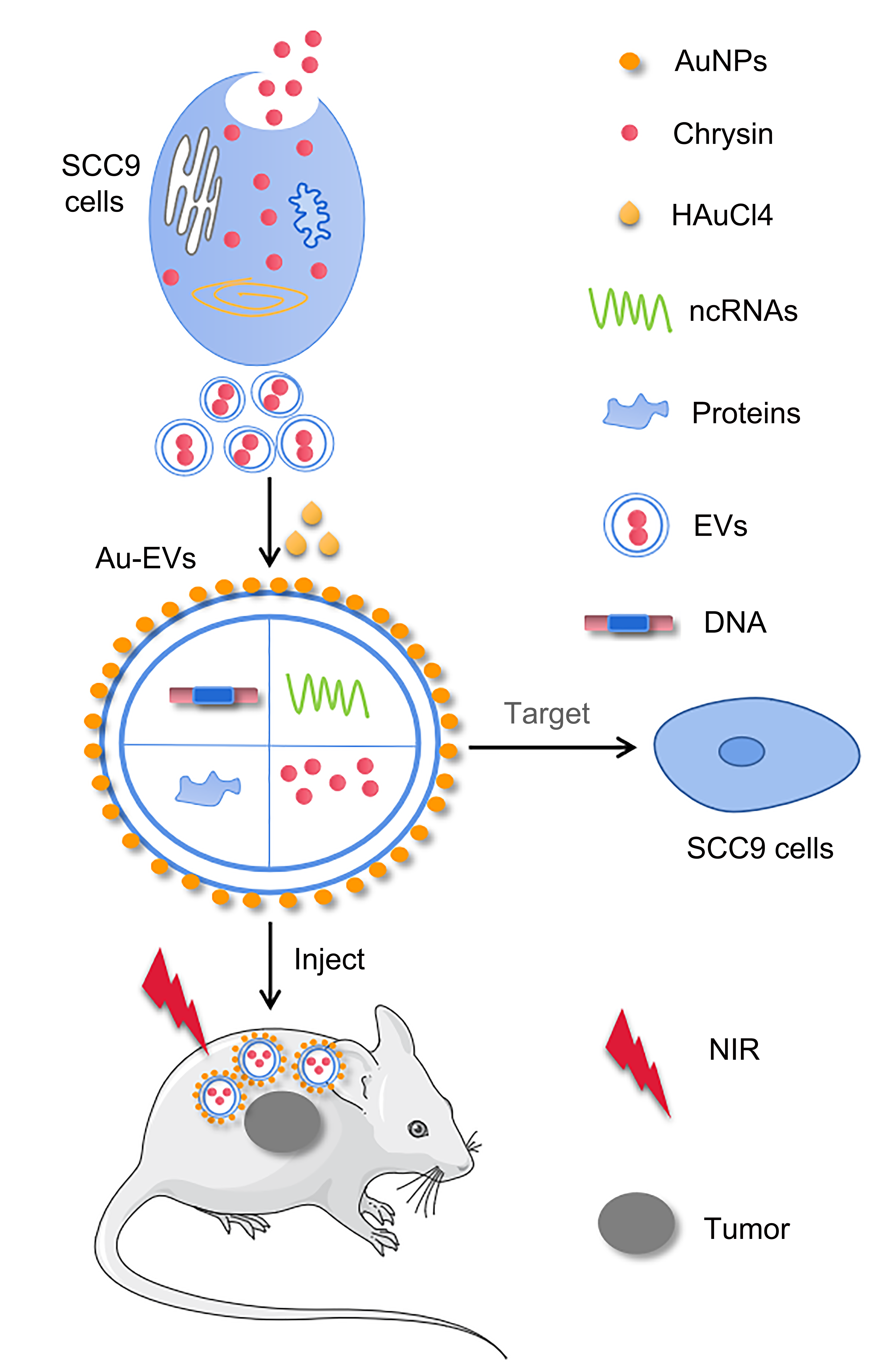

Supplement: Supplementary file 2 [file Image3.TIF]

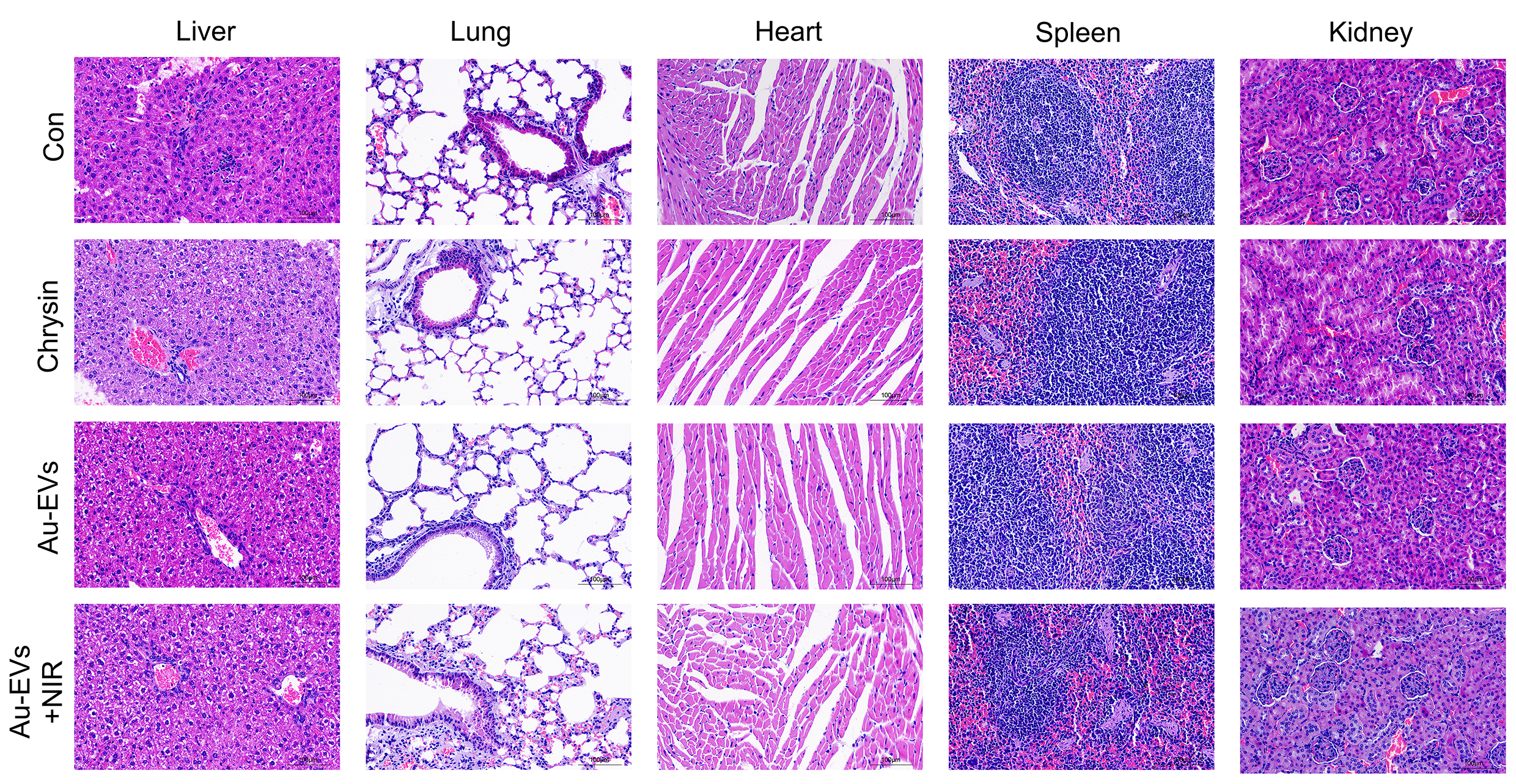

Supplement: Supplementary file 3 [file Image2.TIF]

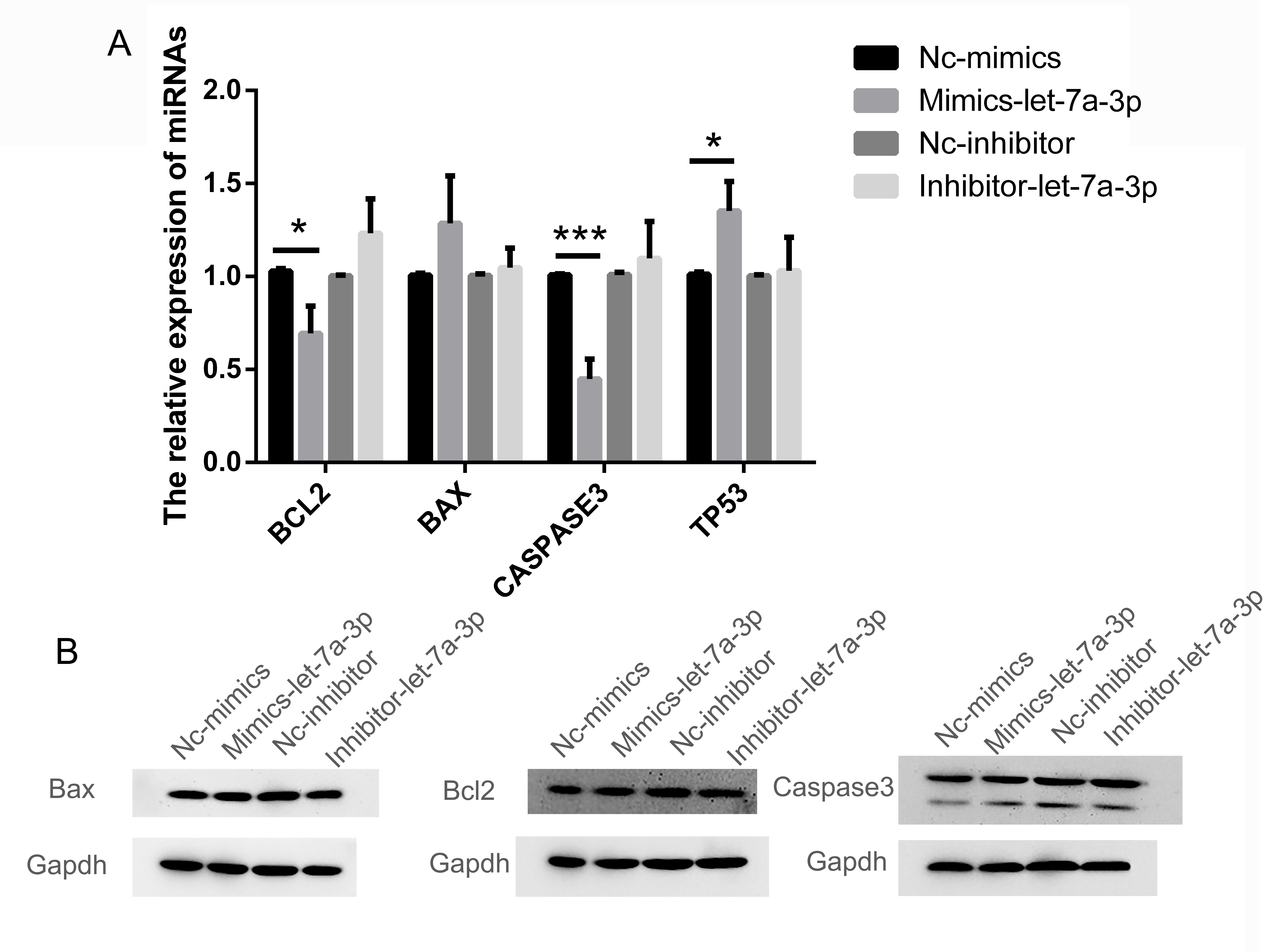

Supplement: Supplementary file 4 [file Image1.TIF]
